# Predicting mental health improvement and deterioration in a large community sample of 11- to 13-year-olds

**DOI:** 10.1007/s00787-019-01334-4

**Published:** 2019-05-03

**Authors:** Miranda Wolpert, Victoria Zamperoni, Elisa Napoleone, Praveetha Patalay, Jenna Jacob, Marjolein Fokkema, Marianne Promberger, Luís Costa da Silva, Meera Patel, Julian Edbrooke-Childs

**Affiliations:** 1 Child Outcomes Research Consortium (CORC), Anna Freud National Centre for Children and Families, 12 Maresfield Gardens, London, NW3 5SU UK; 2 University of Liverpool, Liverpool, UK; 3 University of Leiden, Leiden, The Netherlands

**Keywords:** Improvement, Deterioration, Child mental health, Outcomes

## Abstract

Of children with mental health problems who access specialist help, 50% show reliable improvement on self-report measures at case closure and 10% reliable deterioration. To contextualise these figures it is necessary to consider rates of improvement for those in the general population. This study examined rates of reliable improvement/deterioration for children in a school sample over time. *N* = 9074 children (mean age 12; 52% female; 79% white) from 118 secondary schools across England provided self-report mental health (SDQ), quality of life and demographic data (age, ethnicity and free school meals (FSM) at baseline and 1 year and self-report data on access to mental health support at 1 year). Multinomial logistic regressions and classification trees were used to analyse the data. Of 2270 (25%) scoring above threshold for mental health problems at outset, 27% reliably improved and 9% reliably deteriorated at 1-year follow up. Of 6804 (75%) scoring below threshold, 4% reliably improved and 12% reliably deteriorated. Greater emotional difficulties at outset were associated with greater rates of reliable improvement for both groups (above threshold group: OR = 1.89, *p* < 0.001, 95% CI [1.64, 2.17], below threshold group: OR = 2.23, *p* < 0.001, 95% CI [1.93, 2.57]). For those above threshold, higher baseline quality of life was associated with greater likelihood of reliable improvement (OR = 1.28, *p* < 0.001, 95% CI [1.13, 1.46]), whilst being in receipt of FSM was associated with reduced likelihood of reliable improvement (OR = 0.68, *p* < 0.01, 95% CI [0.53, 0.88]). For the group below threshold, being female was associated with increased likelihood of reliable deterioration (OR = 1.20, *p* < 0.025, 95% CI [1.00, 1.42]), whereas being from a non-white ethnic background was associated with decreased likelihood of reliable deterioration (OR = 0.66, *p* < 0.001, 95% CI [0.54, 0.80]). For those above threshold, almost one in three children showed reliable improvement at 1 year. The extent of emotional difficulties at outset showed the highest associations with rates of reliable improvement.

**Electronic supplementary material:**

The online version of this article (10.1007/s00787-019-01334-4) contains supplementary material, which is available to authorized users.

## Introduction

Up to half of adult mental health difficulties originate in adolescence [1] and the negative impact of children’s mental health difficulties on educational attainment, drug use, criminality, physical health and later workforce involvement and financial difficulties have been widely stressed [2–5]. As many as 1 in 4 adolescents report difficulties at levels likely to indicate significant mental health problems, and the majority have multiple difficulties which may share common aetiology [1, 6, 7]. Despite this, only about one in five children with mental health difficulties currently access mental health support [8]. The majority of those accessing such services have multiple problems [9].

Following the approach pioneered in early intervention in adult mental health in the UK [9], there is increasing interest in considering improvement or deterioration across multiple domains of functioning, rather than just considering outcomes in one domain (e.g. depression or conduct disorder). This can provide a more complete picture of outcomes for children. A key metric proposed for use is overall reliable improvement [10]. This metric involves assessment of change across multiple problem areas such that reliable improvement is said to occur when a score on at least one domain (e.g. low mood) improves enough for it to be considered ‘reliable’ (unlikely to have happened by measurement fluctuation alone) [11], and no score on any other domain (e.g. conduct problems) has reliably deteriorated [12]. Reliable deterioration is taken to occur if the score on any domain deteriorates enough for it to be considered statistically unlikely to have happened by measurement fluctuation alone [11], regardless of whether a score on any other domain has reliably improved.

A small but growing literature has started to consider overall reliable change rates aggregated as above across multiple domains for those accessing child mental health services. The extant literature suggests that around 50% show reliable improvement in their self-reported level of symptoms for at least one of these problems (and not deterioration in any other problem) at the end of treatment, whilst around 10% show deterioration in at least one problem area [13–15]. This is in line with reliable change rates for adults with early-stage anxiety or depression accessing early intervention services in the UK [16].

There is mixed evidence on the stability of psychopathology in childhood and adolescence, with some studies finding temporal stability [17–20] and other studies finding temporal instability [21–23]. There is an existing, albeit patchy, literature on improvement and deterioration in children who do not access help; i.e. any input from a professional, including therapist, counsellor, medical professional, etc. Whilst some studies have focussed on multiple domains (e.g. of children classified as above the clinical cut off at age 9 on any parent reported sub-scale on the Strengths and Difficulties Questionnaire (SDQ), only 41% were still above the clinical cut off at 13 [23]), this literature on what has been termed “spontaneous remission” (i.e. improvement in the absence of a professionally-led intervention) has tended to focus only on one symptom at a time, rather than looking at multiple domains of functioning, with mixed results. In terms of social anxiety, a community sample in Germany (*n* = 2210) found 15% in remission between 14–24 years [24]. In terms of separation anxiety, a US sample found a 23% decline year on year from the age of 10 (*n* = 2384; mean age 14.6 [25]). In terms of depression, a systematic review of five child and adolescent studies found 53% of major depression cases spontaneously remit over a year [26]. In terms of conduct disorder, findings from a 4-year follow-up of a community sample of children in Canada found that of the children who met the criteria for conduct disorder at age ten, 50% did not meet the criteria by age 14 [27]. With regard to ADHD, of 132 children meeting ADHD criteria in grade 1 (ages 6–7), 31% were in remission by grade 4 (ages 9–10) [28]. This is an area of pressing interest if we are to develop the most effective interventions and support for those with mental health problems. Without knowing both (1) expected rates of reliable improvement and (2) the full range of factors related to improvement and deterioration, interpretation of change rates for children in therapy is not possible. In the absence of randomisation, it is hard to disentangle what progress would have occurred without intervention. It is acknowledged that mental health problems may often follow a relapse-remitting course. However, there has been little focus in the literature on how to distinguish change following contact with professional support from regression to the mean (resulting in increased likelihood of movement away from extreme scores) or “spontaneous improvement” (which might otherwise be termed “improvement not mediated by a professional” since it may not be spontaneous but the results of other influences).

The aim of the current research is to identify characteristics predictive of the occurrence of reliable change for both those with and without mental health problems at outset in a large community sample. A secondary aim was to make use of classification trees to help consider and present the key predictors found from the multinomial regressions. Such classification trees allow for the visual investigation of complex relationships between predictors, making the results more accessible to an audience of non-statisticians and in particular allowing for use by clinicians in their frontline practice [29, 30].

## Methods

### Participants and procedure

The present study focused on data collected from 9074 secondary-school students aged 11–12 at baseline and 12–13 at 1-year follow-up from 118 schools [31]. The data corpus included young people from a 3-year longitudinal study and RCT of secondary school students from 75 geographic areas of England conducted between 2008 and 2011. In this study, students completed measures using a secure on-line platform. Cases were deemed eligible for analysis if they had valid data for all the key mental health, socio-demographic, functioning and environment, and treatment variables (described in the Measures section below). 22,359 participants from 204 schools had data at T1, however, only 118 schools participated at T2, restricting the participants to the 118 schools that participated at both T1 and T2. In the 118 schools that participated at both timepoints there were 14,296 participants at T1 and 9074 participants with follow-up at T2 (63% of those with T1 data). Students who were male, eligible for free school meals (FSM) and Black or Other ethnic groups were less likely to have data at follow-up. Students with follow-up had significantly lower T1 scores on the emotional (*M *= 2.61 vs 2.77; *t* = 4.17, *p* < 0.001), conduct (*M *= 1.96 vs 2.27; *t* = 8.86, *p* < 0.001), hyperactivity (*M *= 3.72 vs 3.94; *t* = 5.29, *p* < 0.001), and peer-problem (*M *= 1.81 vs 2.02; *t* = 6.54, *p* < 0.001) subscales, as well as higher impact of their problems than those who dropped out (*M *= 0.72 vs 0.95; *t *= 7.28 all *p *< 0.001). The characteristics of young people in the full sample are displayed in Table 1.

**Table 1:** Characteristics of the full sample

Variable	*N*	Mean	SD
Baseline conduct	9074	1.96	1.88
Baseline emotion		2.61	2.18
Baseline hyperactivity		3.72	2.35
Baseline peer problems		1.81	1.74
Baseline impact		0.72	1.71
Attainment at appropriate level for academic stage		28.29	5.44
School climate		9.44	3.09
Quality of life		25.38	5.50

	*N*	%
Gender		
Male	4363	48.08
Female	4711	51.92
Ethnicity		
White or white British	7142	78.71
Asian or Asian British	882	9.72
Black or black British	550	6.06
Mixed	365	4.02
Other	135	1.49
Special educational needs		
Yes	1931	21.28
No	7143	78.72
Free school meals		
Yes	1644	18.12
No	7430	81.88
Duration		
None	5252	57.88
< 1 mo.	1644	18.12
1–5 mo.	875	9.64
6–12 mo.	370	4.08
>1 yr.	933	10.28
School counsellor		
Yes	1126	12.41
No	7948	87.59
Peer mentor		
Yes	909	10.02
No	8165	89.98
Other help in school		
Yes	1333	14.69
No	7741	85.31

*Mo.* months, *yr.* year

These sample characteristics are in line with national data on students at this age, though the rates of deprivation are higher than nationally.

To provide a comparison with evidence on reliable improvement rates in routine practice [32] cases were split into two sub-samples so that we could better identify reliable improvement in a sample of young people with clinical levels of mental health difficulties for whom accessing specialist service may be appropriate:

1. The “above threshold” (*n *= 2270) sample comprised students who scored above the clinical threshold on at least one of the emotional, hyperactivity, and conduct problems subscales of the SDQ at baseline (see “Measures” section for thresholds used). Cases in this sample had a mean age of 12.1 years (SD 0.6), were 52.7% (*n *= 1196) males, 81.5% (*n *= 1849) from a White ethnic background, and 24.0% (*n *= 544) were eligible for FSM—a measure of deprivation.
2. The “below threshold” (*n *= 6804) sample comprised students who scored in the sub-clinical range on all three subscales of the SDQ at baseline. Cases in this sample had a mean age of 12.1 (SD 0.6), were 46.5% (*n *= 3167) males, 77.4% (*n *= 5263) from a White ethnic background, and 16.2% (*n *= 1100) were eligible for FSM.

This research was granted ethical approval by the UCL research ethics committee, reference: 1530/001.

### Measures

#### Mental health difficulties

The emotional, hyperactivity, and conduct problems subscales of the self-reported Strengths and Difficulties Questionnaire (SDQ) [33] were used as the primary outcome in this study. The SDQ is a screening questionnaire composed of 25 items rated on a scale from 0 (not true) to 2 (certainly true). Items can be totalled into five subscales assessing emotional problems, conduct problems, hyperactivity, peer problems, and pro-social behaviour. The measure also contains a supplementary item assessing self-reported problem duration. An additional five items rated on a scale from 0 (not at all) to 3 (a great deal) which assess the impact of difficulties on daily life are available as a supplement to the SDQ and can be totalled to create a total impact score [33]. Three subscales (emotional, hyperactivity, and conduct problems) were selected as primary outcome as they are in line with the approach used in a national evaluation of outcomes from children’s mental health services, and they represent common mental health difficulties in young people. The remaining SDQ subscales (peer problems and prosocial behaviour) are less clearly related to treatment of mental health difficulties, and thus were used as supporting contextual information rather than primary outcomes [15].

The SDQ is a widely used measure which has shown to be sensitive to change in both clinical and community samples. The measure has good validity and reliability, with Cronbach’s alpha ranging between 0.60 and 0.67 across subscales [34]. In the present study, Cronbach’s alpha values for the subscales were 0.66 (conduct problems), 0.72 (emotional problems), and 0.74 (hyperactivity). Thresholds of 5 (conduct), 6 (emotional), and 7 (hyperactivity) were used to identify students likely to be experiencing clinically significant mental health difficulties (the top 10% of the population in a large UK community sample [35]).

The SDQ has also been found to be a useful tool for screening [36], with use of domains (as opposed to total difficulties score) encouraged particularly when assessing children who fall above the threshold [37]. There are three versions of the measure: self-report, parent-report and teacher-report. While all versions have valuable benefits and applications, use of the self-reported version allows the voice of the child to be highlighted. This is particularly important when considering internalising symptoms, a domain where parent- and teacher-reported views have been demonstrated to be disparate [38, 39].

#### Socio-demographic characteristics

Age, gender, ethnicity and socio-economic status were captured by schools as part of the evaluation data collection. Ethnicity was grouped as follows: White (including White British, Irish, and Other White background), Black or Black British, Asian or Asian British, Mixed, and any other ethnic group. Receipt of FSM was used as an indicator of deprivation.

#### Functioning and environment

Contextual information about student functioning and environment was captured through: the presence of special educational needs, mean attainment score (mean score from English, maths and science assessments taken at 11 years), school climate at baseline, peer problems at baseline (as measured by the SDQ), impact of difficulties at baseline (as measured by the SDQ), duration of problems at baseline (as measured by the SDQ), and quality of life.

School climate was assessed using a seven-item child-reported measure that asked questions related to the perceived quality of relationships and support within the school. Items were rated on a scale from 0 (never) to 2 (always) and summed to create a total “school climate” score. The measure has been shown to have good internal consistency, with a Cronbach’s alpha of 0.79 [31].

Quality of life was assessed using nine items selected from the KIDSCREEN-10, a measure of health-related quality of life. The nine items were rated on a scale from 0 (not at all/never) to 4 (extremely/always). The item excluded from the study focused on parental relationship and home life (“Have your parents treated you fairly?”) as it was beyond the scope of the original evaluation study. The nine-item version of the questionnaire had good internal consistency, with a Cronbach’s alpha of 0.75 [40].

#### Types of support accessed

Students were asked at follow-up if they had received support from a peer mentor, a school counsellor, or any “other” source over the previous year. Students who reported seeking help “a few times” or “more than five times” were classified as having received that form of treatment (peer mentor: *n *= 909; school counsellor: *n *= 1123; other source: *n *= 1333) as this was felt to indicate receipt of substantial support from those sources and those that stated they had not sought help or had only done so “once” were classified as having not received treatment (not received peer mentor support: *n *= 8165; not received school counsellor support: *n *= 7948; not received other source of support: *n *= 7741).

### Analytic strategy

#### Reliable change

To assess improvement in mental health difficulties over time, the Reliable Change Index was used. Individuals were said to have improved if they had achieved reliable improvement on at least one of the emotional, hyperactivity, and conduct problems subscales and not deteriorated on any other scale. Reliable change was calculated using the Reliable Change Index: RCI = *x*_2 _− *x*_1_/*S*_diff_ [11, 41]; a formula for assessing if change in score over time is greater than that which could solely be attributed to measurement error. The Reliable Change Index has been used previously in similar work as a method of assessing meaningful change in routinely collected child mental health data [15].

In this study, scores that changed by three points or more on the emotional subscale [reliable change criterion (RCC) 3.2; *S*_diff_ = 1.61], conduct subscale (RCC 3.3, *S*_diff_ = 1.66) and hyperactivity problems subscale (RCC 3.3, *S*_diff_ = 1.71) were considered to have reliably changed (either in a positive or in a negative direction). We took change to be reliable if the Reliable Change Index was > 1.96. As change across three separate subscales was assessed, and to ensure the improvement, deterioration, and no change categories were mutually exclusive, a case was considered reliably improved if any of the three subscales showed reliable improvement and no subscale showed reliable deterioration. A case was considered reliably deteriorated if any of the three subscales showed reliable deterioration, regardless of whether there was any evidence of reliable improvement on other scales. We additionally examined in the above threshold sample how many young people had reliably improved (or deteriorated) on their subscale that was above cut off at outset.

#### Predicting reliable change

A multinomial logistic regression was performed for each of the samples to model the relationship between the predictors and the occurrence of reliable change (either reliable deterioration, or reliable improvement, with no reliable change as the reference category). Analyses were conducted in R (version 3.3.2) [42], using the multinom function of the nnet package [43]. An alpha level of 0.05 was employed for analysis with the full sample, while subsequent analysis of the above and below threshold samples used a Bonferroni adjusted alpha value of 0.025 to reduce the likelihood of Type 1 error. Predictors were added in stages to a null model that contained only the intercept. Variables known to be associated with the development of mental health difficulties and change in mental health difficulties over time [44–46] were entered in four steps: baseline severity of mental health difficulties, socio-demographic characteristics, functioning and environment. Potential other variables were also included to control for their contribution, such as retrospective report on access to help. Continuous variables were standardised to aid interpretation of regression coefficients such that each unit increase/decrease represents a change of one standard deviation.

#### Modelling outcomes using classification trees

The predictors were entered into classification tree models predicting change (no reliable change, reliable deterioration, and reliable improvement) in the above and below threshold samples. Analysis was conducted using the ctree function of the partykit package [47, 48] which utilises recursive partitioning to divide the sample into groups based on binary splits in the predictor variables. It was felt the classification trees would aid in interpreting the key predictors from the multinomial regressions, and allow for the visual investigation of complex relationships between predictors, making the results more accessible to an audience of non-statisticians [29, 30].

## Results

### Rates of reliable change

Table 2 provides the number and percentages of children and young people in the above and below threshold samples whose scores reliably improved, deteriorated or stayed the same from baseline to follow-up. We additionally examined in the above threshold sample how many young people had reliably improved on their subscale that was above cut off at outset. Here, 341 young people reliably improved on the scale that was above threshold at outset (15% of all those above threshold on at least one subscale, 55% of those above threshold who reliably improved). The remainder, 279, had reliably improved on subscales that were not above threshold at outset (12% of those above threshold on at least one subscale, 45% of those above threshold who reliably improved). 73 reliably deteriorated on the scale that was above threshold at outset (3% of all those above threshold on at least one subscale, 37% of those above threshold who reliably deteriorated). 125 had reliably deteriorated on subscales that were not above threshold at outset (6% of those above threshold on at least one subscale, 63% of those above threshold who reliably deteriorated).

**Table 2:** Reliable change rates in the full, above threshold, and below threshold samples

Samples	Improvement			Deterioration			No change		
	*N*	%	95% CI	*N*	%	95% CI	*N*	%	95% CI
Full (*n *= 9074)	910	10.03	[9.21, 10.86]	1011	11.14	[10.33, 11.97]	7153	78.83	[78.01, 79.66]
Above threshold (*n *= 2270)	620	27.31	[25.29, 29.34]	198	8.72	[6.7, 10.75]	1452	63.96	[61.94, 66]
Below threshold (*n *= 6804)	290	4.26	[3.42, 5.13]	813	11.95	[11.11, 12.81]	5701	83.79	[82.95, 84.66]

*CI* confidence intervals

### Predicting change

Table 3 displays the final models for the above and below threshold samples, which represented a significant improvement on the null model with no predictors and provided a significantly better fit to the data than the models with fewer predictors, based on the deviance test.

**Table 3:** Multinomial regression analysis predicting reliable improvement and deterioration in the above and below threshold samples

	Above threshold		Below threshold	
	Improvement	Deterioration	Improvement	Deterioration
Variable	OR [95% CI]	OR [95% CI]	OR [95% CI]	OR [95% CI]
Baseline conduct	1.67*** [1.49, 1.88]	0.81 [0.67, 0.97]	1.07 [0.93, 1.22]	1.09 [1.00, 1.20]
Baseline emotion	1.89*** [1.64, 2.17]	0.68*** [0.54, 0.85]	2.23*** [1.93, 2.57]	0.82*** [0.75, 0.90]
Baseline hyperactivity	1.11 [0.99, 1.24]	0.75*** [0.63, 0.89]	2.26*** [1.92, 2.67]	0.74*** [0.67, 0.81]
Gender (female)	1.00 [0.81, 1.24]	1.14 [0.82, 1.59]	0.74* [0.57, 0.96]	1.20* [1.00, 1.42]
Ethnicity (other)	1.23 [0.95, 1.58]	0.82 [0.54, 1.25]	1.46** [1.09, 1.97]	0.66*** [0.54, 0.80]
FSM (yes)	0.68** [0.53, 0.88]	1.25 [0.88, 1.77]	1.07 [0.77, 1.48]	1.19 [0.97, 1.45]
SEN (yes)	1.12 [0.86, 1.44]	1.01 [0.68, 1.50]	0.80 [0.56, 1.14]	0.92 [0.74, 1.15]
Attainment at appropriate level for academic stage	1.12 [0.99, 1.26]	1.12 [0.92, 1.35]	0.87 [0.75, 1.00]	0.93 [0.85, 1.02]
School climate	0.93 [0.83, 1.04]	1.05 [0.88, 1.25]	1.11 [0.97, 1.28]	0.96 [0.88, 1.05]
Quality of life	1.28*** [1.13, 1.46]	0.78** [0.64, 0.95]	1.11 [0.95, 1.29]	0.93 [ [0.85, 1.03]
Baseline peer problems	1.02 [0.90, 1.15]	0.97 [0.80, 1.17]	1.11 [0.97, 1.26]	1.01 [0.93, 1.10]
Baseline impact	1.00 [0.88, 1.13]	1.02 [0.83, 1.26]	1.00 [0.88, 1.14]	1.02 [0.94, 1.10]
Duration (< 1 month)	0.94 [0.70, 1.25]	0.53** [0.33, 0.85]	0.64** [0.45, 0.91]	1.14 [0.91, 1.41]
Duration (1–5 months)	0.96 [0.70, 1.32]	0.41** [0.23, 0.74]	0.73 [0.47, 1.14]	1.05 [0.77, 1.44]
Duration (6–12 months)	0.80 [0.53, 1.21]	0.77 [0.42, 1.44]	0.76 [0.39, 1.50]	1.45 [0.93, 2.26]
Duration (>1 year)	0.69 [0.50, 0.96]	0.84 [0.53, 1.32]	0.78 [0.49, 1.24]	1.14 [0.82, 1.59]
School counsellor (yes)	0.76 [0.57, 1.02]	1.14 [0.76, 1.71]	0.64 [0.39, 1.04]	1.78*** [1.39, 2.29]
Peer mentor (yes)	0.67* [0.49, 0.93]	1.32 [0.86, 2.02]	1.26 [0.76, 2.08]	1.41** [1.08, 1.86]
Other help (yes)	0.89 [0.67, 1.16]	1.83** [1.24, 2.70]	0.63 [0.41, 1.00]	2.03*** [1.61, 2.55]
McFadden *R*^2^	0.7		0.8	

Continuous variables have been standardised

*OR* odds ratio, *CI* confidence intervals, *FSM* free school meal, *SEN* special educational needs

*p < 0.025, **p < 0.01, ***p < 0.001

For the above threshold sample, the full model AIC was 3686; the null model AIC was 3877 (LR = 266.93, *p *< 0.001; all likelihood ratios comparing to the full model using R’s anova() function). For the reduced model without the retroactive report on access to help (school counsellor, peer mentor, other), the AIC was 3724 (LR = 50.54, *p *< 0.001); for the reduced model including only baseline severity on the subscales, gender, ethnicity, and eligibility for school meals the AIC was 3744 (LR = 110.90, *p* < 0.001); for the reduced model with only baseline severity, the AIC was 3757 (LR = 135.38, *p *< 0.001).

For the below threshold sample, the full model AIC was 6803; the null model AIC was 7305 (LR = 578.34, *p *< 0.001). For the reduced model without reported access to help, AIC was 6926 (LR = 135.14, *p* < 0.001). For the model including baseline severity, gender, ethnicity, eligibility for school meals, the AIC was 6938 (LR = 187.38, *p *<0.001). For the model including only baseline severity, the AIC was 6962 (LR = 222.97, *p* < 0.001).

### Baseline severity

In both samples students reporting higher baseline emotional problems were significantly more likely to reliably improve (above threshold sample: OR = 1.89, *p* < 0.001, 95% CI [1.64, 2.17], below threshold sample: OR = 2.23, *p* < 0.001, 95% CI [1.93, 2.57]), and significantly less likely to reliably deteriorate (above threshold sample: OR = 0.68, *p* < 0.001, 95% CI [0.54, 0.85], below threshold sample: OR = 0.82, *p* < 0.001, 95% CI [0.75, 0.90]).

In the above threshold sample, higher baseline conduct problems were also significantly associated with an increased likelihood of reliable improvement (OR = 1.67, *p* < 0.001, 95% CI [1.49, 1.88]), and higher baseline hyperactivity problems were significantly associated with decreased likelihood of reliable deterioration (OR = 0.75, *p* < 0.001, 95% CI [0.63, 0.89]). In the below threshold sample, higher baseline hyperactivity problems were significantly associated with increased likelihood of reliable improvement (OR = 2.26, *p* < 0.001, 95% CI [1.92, 2.67]) and decreased likelihood of reliable deterioration (OR = 0.74, *p* < 0.001, 95% CI [0.67, 0.81]).

### Socio-demographic characteristics

In the above threshold sample, students in receipt of FSM were significantly less likely to reliably improve (OR = 0.68, *p* < 0.01, 95% CI [0.53, 0.88]). The below threshold sample did not show any significant relationship between receipt of FSM and outcome.

In the below threshold sample, students from any other ethnic background (as compared to White students), were significantly less likely to have reliably improved (OR = 1.46, *p* < 0.01, 95% CI [1.09, 1.97]), and more likely to have reliably deteriorated (OR = 0.66, *p* < 0.001, 95% CI [0.54, 0.80]). Female students were also significantly less likely to have reliably improved (OR = 0.74, *p* < 0.025, 95% CI [0.57, 0.96]), and more likely to have reliably deteriorated (OR = 1.20, *p* < 0.025, 95% CI [1.00, 1.42]). The above threshold sample did not show any significant relationships between ethnicity or gender and outcome.

### Functioning and environment

In the above threshold sample, students with a higher baseline quality of life were significantly more likely to reliably improve (OR = 1.28, *p* < 0.001, 95% CI [1.13, 1.46]), and significantly less likely to reliably deteriorate (OR = 0.78, *p* < 0.01, 95% CI [0.64, 0.95]). In the below threshold sample no significant relationship between quality of life and outcome was found.

### Types of support accessed

In the above threshold sample those who were in receipt of peer mentoring support were significantly less likely to reliably improve (OR = 0.67, *p* < 0.025, 95% CI [0.49, 0.93]).

In the below threshold sample, higher levels of reliable deterioration were associated with receipt of any of the three forms of support; counsellor (OR = 1.78, *p* < 0.001, 95% CI [1.39, 2.29]), peer mentor (OR = 1.41, *p* < 0.01, 95% CI [1.08, 1.86]), and “other” (OR = 2.03, *p* < 0.001, 95% CI [1.61, 2.55]).

### Classification trees

**Fig. 1:**
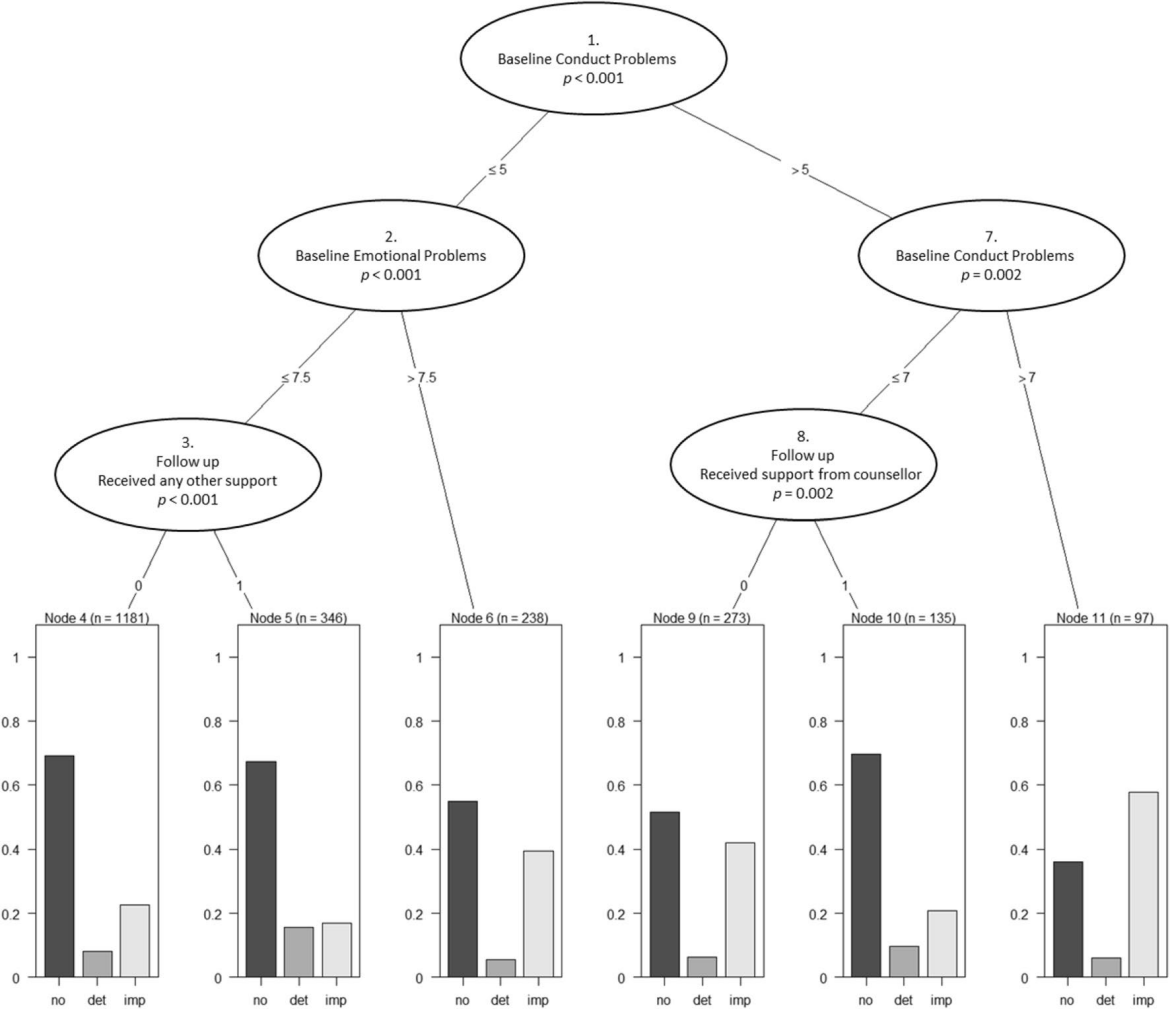
Three-level regression tree predicting reliable improvement, reliable deterioration, and no reliable change in the above threshold sample. *No* no reliable change, *det* reliable deterioration, *imp* reliable improvement. For categorical variables (3, and 8), 1 = yes, 0 = no

**Fig. 2:**
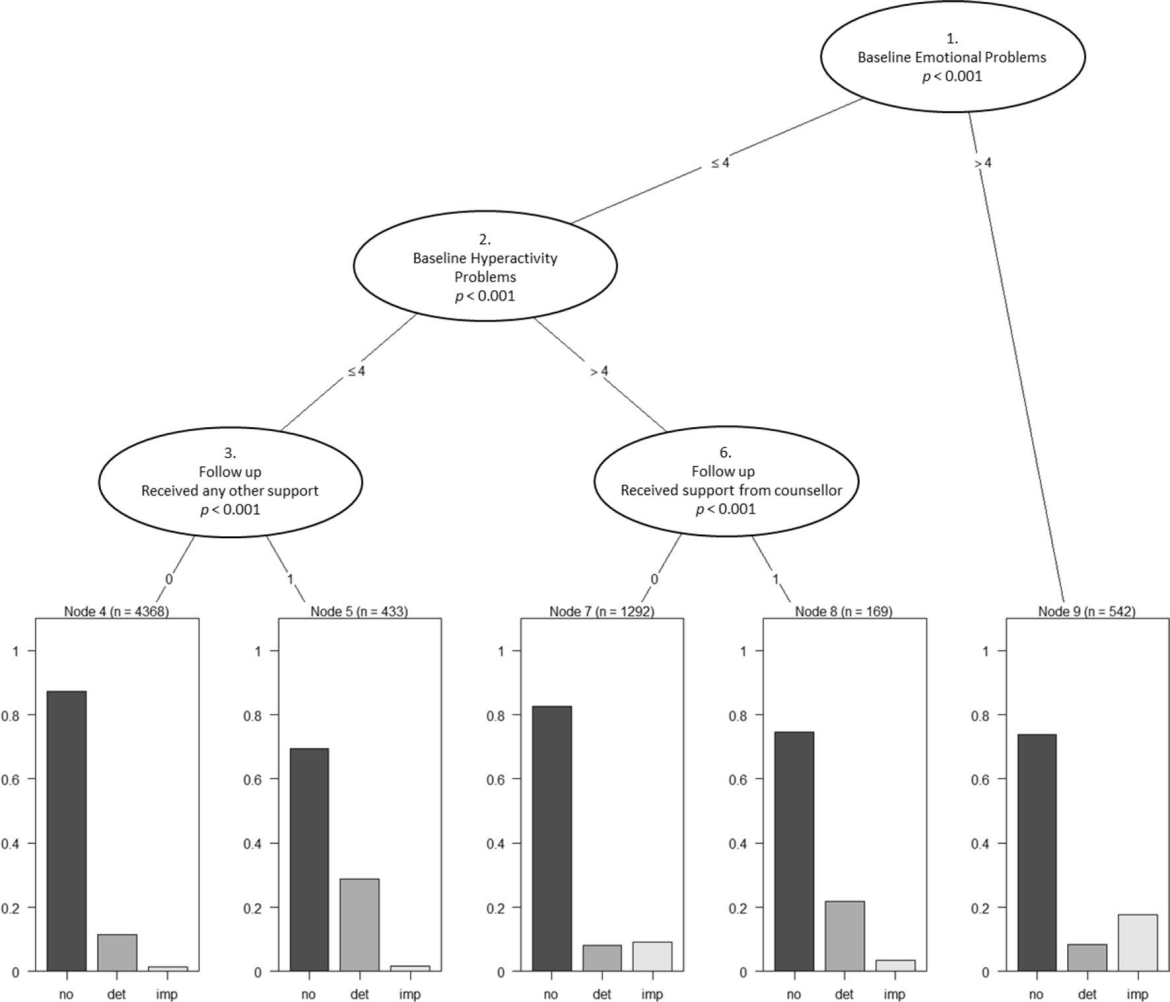
Three-level regression tree predicting reliable improvement, reliable deterioration, and no reliable change in the below threshold sample. *No* no reliable change, *det* reliable deterioration, *imp* reliable improvement. For categorical variables (3, and 6), 1 = yes, 0 = no

All predictors were included as potential predictors in the classification trees for the above and below threshold samples (full sample results are available in supplementary materials). The trees were restricted to three levels as this was felt to be the level of complexity of the tree at which both appropriate level of detail and interpretability of the tree were optimised. The trees are presented in Figs. 1 and 2 (trees up to four levels are presented in the supplementary material).

In the above threshold sample, the classification tree suggested the most important factor in the type of outcome was the baseline severity of conduct problems, with young people reporting scores of greater than seven being most likely to show reliable improvement than deterioration or no change. Among those young people with baseline conduct problem scores between six and seven, those that did not report having seen a counsellor for support were substantially more likely to reliably improve than those that did report having seen a counsellor for support.

Among young people with baseline conduct problems of less than six, and emotional problems of more than 7.5, there was a greater likelihood of improvement compared to those with lower values for emotional problems. For those with emotional scores of 7.5 or lower, those who reported receiving any other form of support showed a higher likelihood of deterioration and lower likelihood of improvement.

In the below threshold sample, the highest chance of reliable improvement was observed in the subgroup with baseline emotional problem scores of greater than four. Young people with scores of four or less for both baseline emotional problems and hyperactivity were more likely to deteriorate if they reported having received any other form of support, as were those with hyperactivity scores greater than four who reported receiving support from a counsellor.

## Discussion

### Children above threshold at outset

All the different analyses undertaken highlighted the impact of baseline severity as the key association with change (the more severe at outset, the more improvement seen). The classification tree, for example, highlights that those with a conduct disorder score above seven on the SDQ are the most likely to show reliable improvement a year later. This highlights the likely impact of regression to the mean and the need for practitioners and researchers to take this into account in considering the interpretation of change over time.

A higher baseline quality of life was associated with a greater likelihood of improvement, and a reduced likelihood of deterioration, whilst being in receipt of FSM was associated with a reduced likelihood of improvement. This finding is in line with evidence that mental health and wellbeing are distinct constructs [49]. This raises the possibility that children who report a higher quality of life, despite mental health difficulties, may be more able to find ways to address or manage their difficulties, whilst those who are in contexts of greatest social deprivation may find it hardest to find ways to manage or overcome such difficulties. This might suggest the possibility of focussing on improving general quality of life for those with mental health difficulties to help them to develop resilience and access resources that help address their difficulties. Targeting resources at those living in the greatest circumstances of deprivation would also be a possibility given evidence on the association between socio-economic deprivation and increased levels of mental health difficulties [50, 51].

Overall nearly one in three children above threshold at time one will have shown reliable improvement at time two. This is lower than evidence from specialist services that one in two children above threshold at time one show reliable improvement at time two [32]. The findings of the present research are also in line with previous studies showing 15–50% spontaneous remission of mental health difficulties in young people [24–28]. The majority of these have not have accessed specialist support. There was a relatively short time period between the baseline and follow-up, and a longer interval may mean even greater change would be expected. Children who reported accessing specialist support showed reduced likelihood of improvement (peer mentoring in school), increased the likelihood of deterioration (contact with specialist support outside school) or no association (counselling). The association between reduced improvement/increased deterioration and access to help is most likely explained by the fact that the lack of improvement/deterioration prompts contact with services, but it should be noted that the amount of contact (“several meetings” or “more than 5 contacts”) is close to the total number of contacts children are likely to receive when accessing services [15]. Forms of self-help or community support that lead to what has until now been termed “spontaneous improvement” is an area in need of further attention.

The fact that most young people who exhibited reliable improvement often did so on subscales that were not above the threshold may reflect the stability of difficulties in a single domain but may also reflect complex interdependencies and interplay between different mental health issues in young people, thought to be related to a common factor of distress [52].

### Children below threshold at outset

Again, the different analyses undertaken highlighted the impact of baseline severity as the key association with change (the more severe at outset, the more improvement seen). The classification tree for those below threshold found that those with an emotional disorder score above four on the SDQ are the most likely to show reliable improvement a year later. Given the lack of extreme scores at outset, and a possible floor effect in that scores less than three at outset could not demonstrate reliable improvement (of three points), there was far less change in this group; of those below threshold at the outset only 5% reliably improved and 11% reliably deteriorated.

Being female was associated with decreased likelihood of improvement and increased likelihood of deterioration, whereas being from any other ethnic background (as compared to White students) was associated with increased likelihood of improvement and decreased likelihood of deterioration. This is in line with other literature on risk factors for mental health problems being raised for females and reduced for some minority ethnic groups [6], suggesting the potential to focus on girls for prevention initiatives. However, the low rates of deterioration overall in this group might suggest the benefits of focussing on those with emerging mental health problems.

Children who accessed any form of speciality mental health (peer mentoring, counselling, help outside school) were more likely to show deterioration than those who did not access this support. As with those above the threshold, this association is most likely explained by the fact that deterioration prompts contact with services. A limitation of the present study is that reliable data on contact with clinical services was not available and whether peer mentoring can legitimately be considered a form of specialist support is debatable.

Clearly, a key limitation of this research is the fact that the analyses are all correlational and no direction of causality can be inferred. The explorations above should thus be taken as starting points for developing hypotheses and further studies. The lack of randomisation means that it is not possible to disentangle spontaneous remission or regression from the mean from the impact of services. A further limitation is the fact that the data were based on self-report. For example, the children’s report of their access to help may have been inaccurate in key ways that are not possible to determine from the findings. In particular, evidence suggests that it may be especially difficult to accurately self-report on externalizing problems [38, 39]. Moreover, the present sample was different from the full sample in a number of ways. In particular, the following groups were under-represented at follow-up: boys, students with ethnic categories of Black or other, students eligible for free school meals and those with higher mental health problems at outset. Interestingly the group with follow up data tended to have a higher impact of problems on their life at time one even if they had lower levels of problems overall. These skews in the data clearly affects the generalisability of the results and further research is needed to consider change in these under-represented groups in particular.

However, whilst acknowledging these real limitations, we do feel this paper adds to the literature by being a first step to exploring potential predictors of mental health improvement and deterioration for children. There is a particular emphasis on considering how to think about the difference between change that can be attributed to the impact of service provision and that which can be attributed to other factors. In addition, we have presented the importance of considering the child in terms of multiple domains, rather than only considering improvement in one area of functioning. The limitation of only exploring one domain of functioning is that it simplifies the complex nature of mental health difficulties whereby comorbidity is common. The multi-domain approach taken in the present research adds a multi-layered perspective to considering the improvement of symptoms, which then feeds into implications for schools, policy and practice in terms of both support provision and consideration about how children might improve without such professional input.

In considering improvement in multiple domains, we hope we have started a conversation about how to consider the best ways to disentangle regression to the mean from spontaneous improvement which occurs as a result of the impact of different forms of self-care or support. More research is needed to take this forward.

## Conclusion

Overall nearly one in three children above threshold at time one will have shown reliable improvement at time two. The majority of these have not have accessed specialist support. This research is a first step to trying to consider factors associated with improvement and deterioration in mental health problems in children and found associations with a range of factors, with the strongest predictor of improvement being high levels of difficulties at outset. Classification trees may be one way to help clinicians take these factors into account in their clinical practice.

## Electronic supplementary material

Below is the link to the electronic supplementary material.

Supplementary material 1 (DOCX 528 kb)

## Abbreviation

FSM: Free school meals
