# Supplementary material for: Predicting mental health improvement and deterioration in a large community sample of 11- to 13-year-olds
Source: Eur Child Adolesc Psychiatry. 2019 May 3;29(2):167–78. doi: 10.1007/s00787-019-01334-4 (PMC7024693; doi:10.1007/s00787-019-01334-4)
Supplement: Supplementary file 1 — Supplementary material 1 (DOCX 528 kb) [file 787_2019_1334_MOESM1_ESM.docx]

Table S1

*Multinomial Regression Analysis Predicting Reliable Improvement and Deterioration in the Full Sample*

|  | Step 1 |  | Step 2 | |  | | Step 3 | |  | | Step 4 | |  | |
| --- | --- | --- | --- | --- | --- | --- | --- | --- | --- | --- | --- | --- | --- | --- |
|  | Improvement | Deterioration | | Improvement | | Deterioration | | Improvement | | Deterioration | | Improvement | | Deterioration |
| Variable | OR  [95% CI] | OR  [95% CI] | | OR  [95% CI] | | OR  [95% CI] | | OR  [95% CI] | | OR  [95% CI] | | OR  [95% CI] | | OR  [95% CI] |
| Baseline Conduct | 1.57***  [1.45, 1.69] | 1.14**  [1.05, 1.24] | | 1.57***  [1.44, 1.70] | | 1.15**  [1.05, 1.25] | | 1.62***  [1.48, 1.76] | | 1.08  [0.98, 1.18] | | 1.67***  [1.53, 1.83] | | 0.99  [0.90, 1.09] |
| Baseline Emotion | 1.93***  [1.80, 2.07] | 0.96  [0.89, 1.03] | | 1.96***  [1.83, 2.11] | | 0.94  [0.87, 1.01] | | 2.25***  [2.06, 2.47] | | 0.84***  [0.77, 0.92] | | 2.30***  [2.10, 2.52] | | 0.80***  [0.73, 0.88] |
| Baseline Hyperactivity | 1.56***  [1.43, 1.71] | 0.79***  [0.76, 0.86] | | 1.58***  [1.44, 1.72] | | 0.78***  [0.72, 0.85] | | 1.66***  [1.51, 1.83] | | 0.76***  [0.69, 0.83] | | 1.67***  [1.52, 1.84] | | 0.75***  [0.68, 0.82] |
| Gender (Female) |  |  | | 0.91  [0.77, 1.06] | | 1.11  [0.97, 1.28] | | 0.90  [0.77, 1.06] | | 1.16*  [1.00, 1.33] | | 0.90  [0.77, 1.06] | | 1.17*  [1.01, 1.35] |
| Ethnicity (Other) |  |  | | 1.32**  [1.10, 1.59] | | 0.71***  [0.60, 0.84] | | 1.32**  [1.09, 1.59] | | 0.69***  [0.58, 0.82] | | 1.30**  [1.08, 1.58] | | 0.71***  [0.59, 0.84] |
| FSM (Yes) |  |  | | 0.77**  [0.63, 0.93] | | 1.32**  [1.11, 1.56] | | 0.77**  [0.63, 0.94] | | 1.26**  [1.06, 1.49] | | 0.79*  [0.64, 0.96] | | 1.19*  [1.00, 1.42] |
| SEN (Yes) |  |  | |  | |  | | 0.96  [0.78, 1.18] | | 1.02  [0.85, 1.24] | | 1.01  [0.83, 1.25] | | 0.94  [0.77, 1.14] |
| Key Stage Attainment |  |  | |  | |  | | 1.00  [0.91, 1.09] | | 0.91*  [0.84, 0.99] | | 0.98  [0.89, 1.07] | | 0.96  [0.88, 1.04] |
| School Climate |  |  | |  | |  | | 0.94  [0.87, 1.03] | | 0.99  [0.91, 1.07] | | 0.96  [0.88, 1.04] | | 0.96  [0.89, 1.04] |
| Quality of Life |  |  | |  | |  | | 1.26***  [1.14, 1.39] | | 0.89**  [0.81, 0.97] | | 1.26***  [1.14, 1.39] | | 0.89**  [0.81, 0.97] |
| Baseline Peer  Problems |  |  | |  | |  | | 1.03  [0.95, 1.13] | | 1.03  [0.95, 1.12] | | 1.05  [0.97, 1.15] | | 1.00  [0.92, 1.09] |
| Baseline Impact |  |  | |  | |  | | 0.94  [0.86, 1.02] | | 1.03  [0.95, 1.11] | | 0.95  [0.87, 1.03] | | 1.00  [0.92, 1.08] |
| Duration  (< 1 mo.) |  |  | |  | |  | | 0.93  [0.75, 1.15] | | 1.04  [0.86, 1.27] | | 0.92  [0.74, 1.14] | | 1.04  [0.86, 1.26] |
| Duration  (1-5 mo.) |  |  | |  | |  | | 0.98  [0.76, 1.26] | | 0.95  [0.72, 1.24] | | 0.99  [0.77, 1.28] | | 0.89  [0.67, 1.16] |
| Duration  (6-12 mo.) |  |  | |  | |  | | 0.89  [0.62, 1.26] | | 1.38  [0.97, 1.96] | | 0.89  [0.62, 1.26] | | 1.31  [0.92, 1.87] |
| Duration  (>1 yr.) |  |  | |  | |  | | 0.72*  [0.55, 0.94] | | 1.25  [0.97, 1.62] | | 0.73*  [0.56, 0.96] | | 1.18  [0.91, 1.53] |
| School Counsellor (Yes) |  |  | |  | |  | |  | |  | | 0.75*  [0.58, 0.96] | | 1.59***  [1.28, 1.96] |
| Peer Mentor (Yes) |  |  | |  | |  | |  | |  | | 0.78  [0.59, 1.03] | | 1.34**  [1.07, 1.69] |
| Other Help (Yes) |  |  | |  | |  | |  | |  | | 0.79*  [0.62, 1.00] | | 1.96***  [1.61, 2.39] |
| McFadden R^2^ | 0.10 | | | 0.10 | | | | 0.11 | | | | 0.12 | | |

*Notes*. Continuous variables have been standardized. OR = Odds Ratio; CI = Confidence Intervals; FSM = Free School Meal; SEN = Special Educational Needs.

* p < .05, ** p < .01, *** p < .001


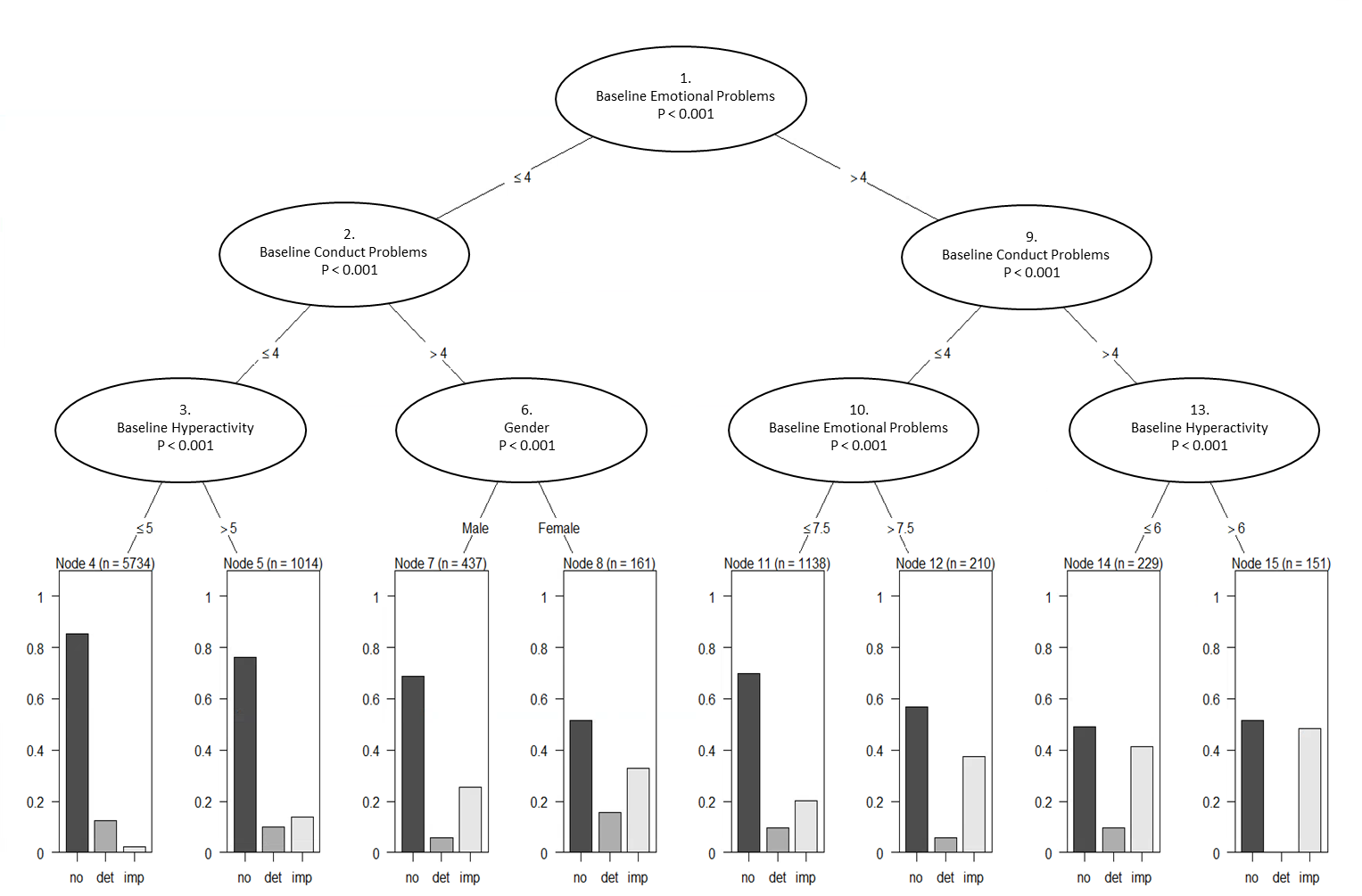


*Figure S1*. Three-level regression tree predicting reliable improvement, reliable deterioration, and no reliable change in the full sample

*Note*. No = no reliable change; det. = reliable deterioration; imp = reliable improvement.


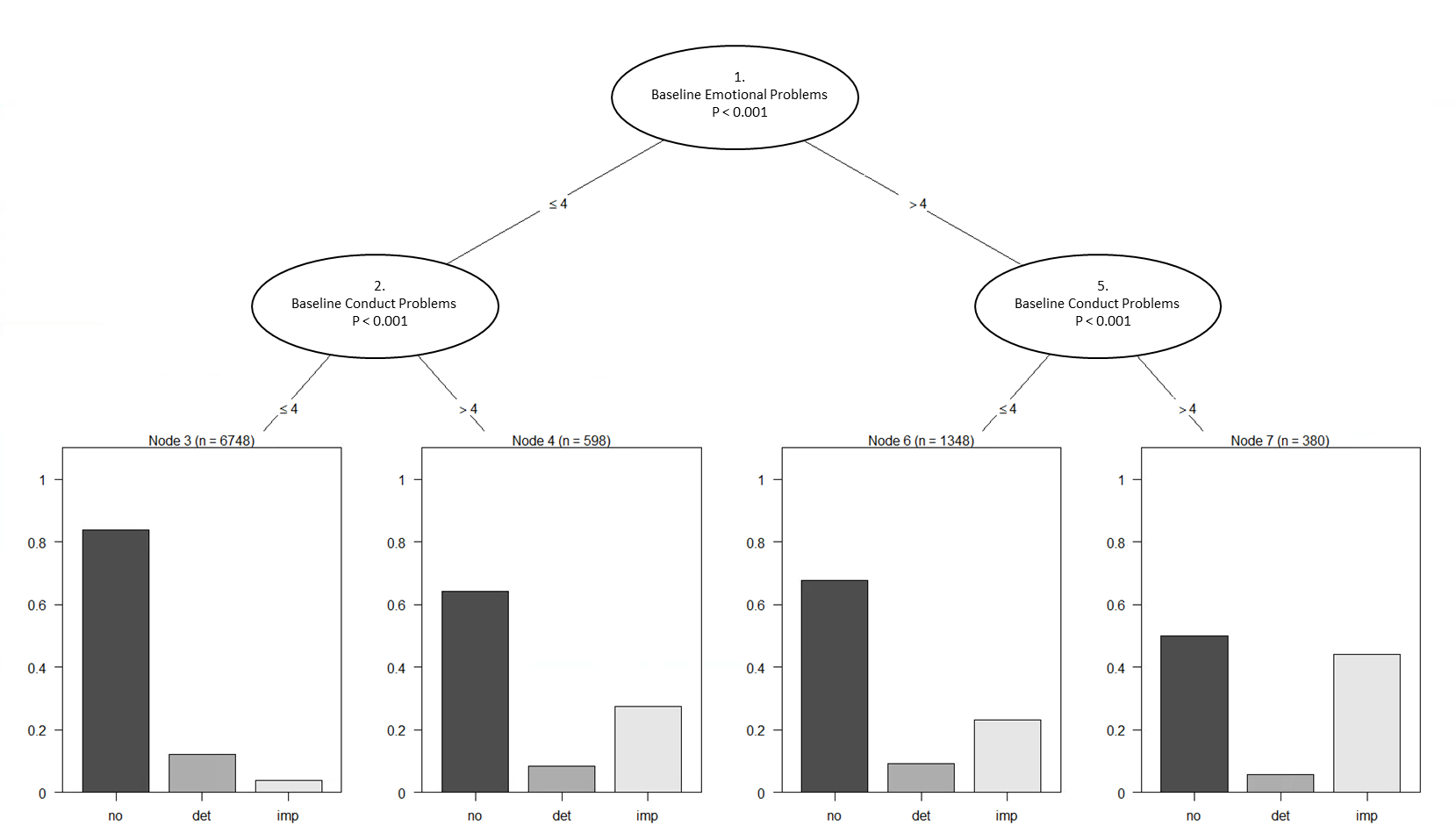


*Figure S2*. Two-level regression tree predicting reliable improvement, reliable deterioration, and no reliable change in the full sample

*Note*. No = no reliable change; det. = reliable deterioration; imp = reliable improvement.


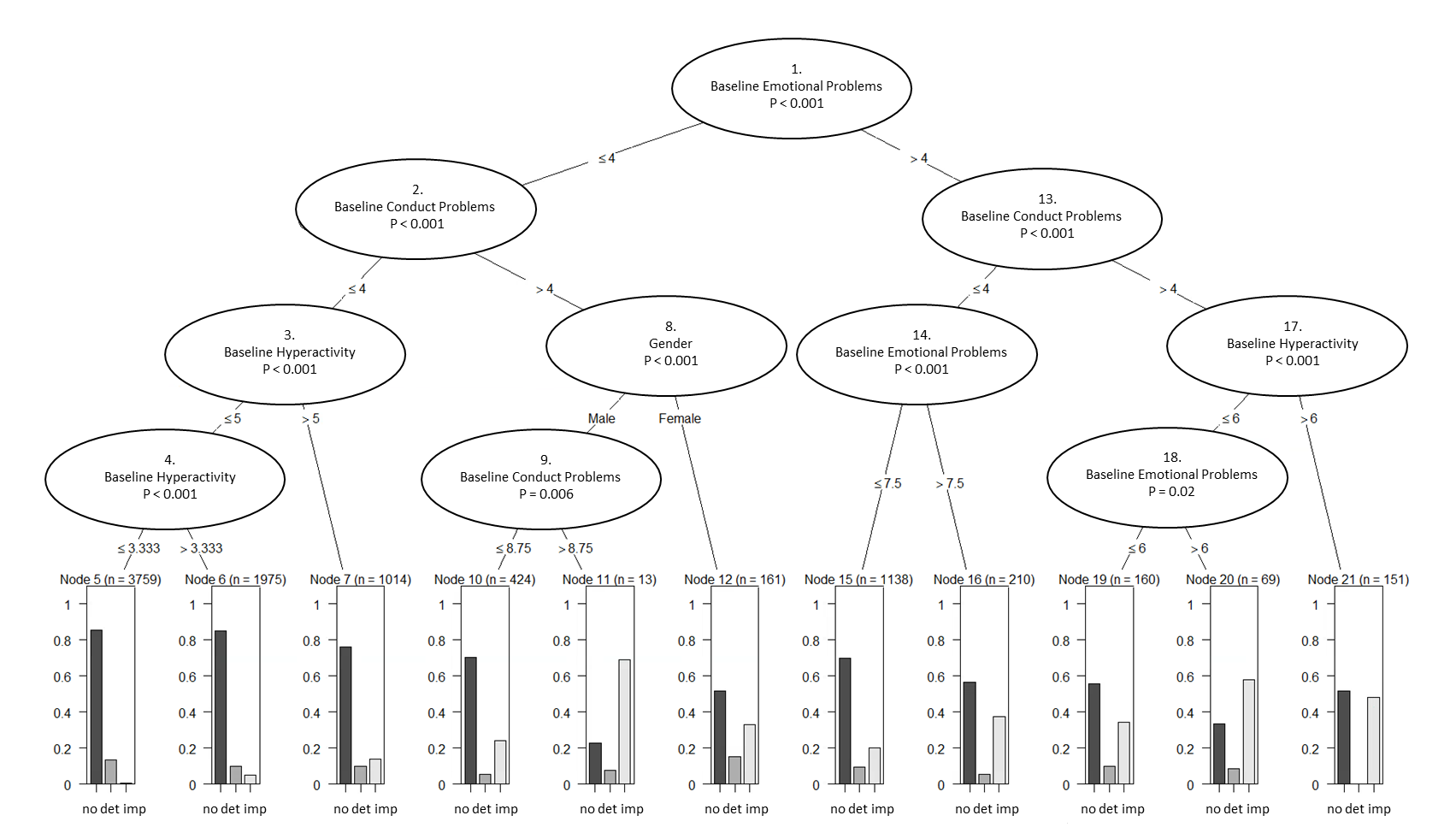


*Figure S3.* Four-level regression tree predicting reliable improvement, reliable deterioration, and no reliable change in the full sample

*Note*. No = no reliable change; det. = reliable deterioration; imp = reliable improvement.
